# Computational drug repurposing in Parkinson’s disease: Omaveloxolone and cyproheptadine as promising therapeutic candidates

**DOI:** 10.3389/fphar.2025.1539032

**Published:** 2025-04-08

**Authors:** Xin Guo, Jie Wang, Hongyang Fan, Wanying Tao, Zijing Ren, Xingyue Li, Suyu Liu, Peiyang Zhou, Yingzhu Chen

**Affiliations:** ^1^ Department of Geriatric Neurology, Northern Jiangsu People’s Hospital Affiliated to Yangzhou University, Yangzhou, Jiangsu, China; ^2^ Department of Neurology, Xiangyang No.1 People’s Hospital, Hubei University of Medicine, Xiangyang, China; ^3^ Department of Critical Care Medicine, Department of Emergency Medicine, Xiangyang Central Hospital, Affiliated Hospital of Hubei University of Arts and Science, Xiangyang, Hubei, China; ^4^ Medical College, Nanjing University, Nanjing, China

**Keywords:** computational repurposing, Parkinson’s disease, Omaveloxolone, cyproheptadine, MAPK/NF κ B signaling pathway, Keap1-Nrf2/ARE signaling pathway

## Abstract

**Background:** Parkinson's disease (PD), a prevalent and progressive neurodegenerative disorder, currently lacks effective and satisfactory pharmacological treatments. Computational drug repurposing represents a promising and efficient strategy for drug discovery, aiming to identify new therapeutic indications for existing pharmaceuticals.

**Methods:** We employed a drug-target network approach to computationally repurpose FDA-approved drugs from databases such as DrugBank. A literature review was conducted to select candidates not previously reported as pharmacoprotective against PD. Subsequent in vitro evaluation utilized Cell Counting Kit-8 (CCK8) assays to assess the neuroprotective effects of the selected compounds in the SH-SY5Y cell model of Parkinson's disease induced by 1-methyl-4-phenylpyridinium (MPP+). Furthermore, an in vivo mouse model of Parkinson's disease induced by 1-methyl-4-phenyl-1,2,3,6-tetrahydropyridine (MPTP) was developed to investigate the mechanisms of action and therapeutic potential of the identified drug candidates.

**Results:** Our approach identified 176 drug candidates, with 28 selected for their potential anti-Parkinsonian effects and lack of prior PD-related reporting. CCK8 assays showed significant neuroprotection in SH-SY5Y cells for Omaveloxolone and Cyproheptadine. In the MPTP-induced mouse model, Cyproheptadine inhibited interleukin-6 (IL-6) expression and prevented Tyrosine Hydroxylase (TH) downregulation via the MAPK/NFκB pathway, while Omaveloxolone alleviated TH downregulation, potentially through the Kelch-like ECH-associated protein 1 (KEAP1)-NF-E2-related factor 2 (Nrf2)/antioxidant response element (ARE) pathway. Both drugs preserved dopaminergic neurons and improved neurological deficits in the PD model.

**Conclusion:** This study elucidates potential drug candidates for the treatment of Parkinson's disease through the application of computational repurposing, thereby underscoring its efficacy as a drug discovery strategy.

## 1 Introduction

Parkinson’s disease (PD) ranks as the second most prevalent neurodegenerative disorder globally. With the advancement of global aging, there is a concomitant rise in the absolute number of individuals affected by Parkinson’s disease, with an estimated lifetime risk of approximately 5% (Ben-Shlomo et al., 2024). The primary clinical manifestations of PD include resting tremor, altered muscle tone, and bradykinesia. These symptoms are linked to the degeneration of dopaminergic neurons and the accumulation of protein aggregates containing α-synuclein (α-syn) within dopamine neurons, known as Lewy bodies (Morris et al., 2024). Although there have been some advances in the treatment of Parkinson’s disease patients in recent years, such as dopamine replacement therapy and deep brain stimulation for symptom control, these treatments still do not provide significant improvement in the quality of life of Parkinson’s disease patients due to the unsustainable substitution of medications and the inconvenience of surgery required for deep brain stimulation (Hill et al., 2023). However, little is known about how to delay the death of dopaminergic neurons in Parkinson’s patients, and there is an urgent need to develop new and effective Parkinson’s drugs.

The traditional approach to drug design is both time-consuming and costly, spanning from initial development to clinical application. Enhancing research and development productivity necessitates minimizing attrition rates and cycle times in clinical trials (Paul et al., 2010). To achieve a reduction in both the drug development timeline and associated costs, as well as to streamline the clinical trial process, drug repurposing emerges as a viable drug discovery strategy. This approach facilitates the identification of new therapeutic indications for already approved drugs, thereby mitigating the risk of failure due to drug toxicity. A study in 2015 demonstrated that screened nine non-Parkinson’s disease drug candidates from approved drugs by a new bidirectional drug repositioning method and developed an On-target ratio (OTR) parameter, which was higher than that of known Parkinson’s drugs for all nine drugs (Rakshit et al., 2015). Using an artificial intelligence (AI)-based drug repurposing strategy, Jae-Bong Kim et al. found that the antiviral drugs efavirenz and nevirapine attenuated α-syn proliferation and neuroinflammation in PD mice (Kim et al., 2024). There is also an end-to-end knowledge graph-based computational drug repurposing method called EKGDR that predicts the probability of interaction for each drug-disease pair, yielding the top 20 drug candidates for Alzheimer’s and Parkinson’s diseases (Tayebi and BabaAli, 2024). Computational repurposing represents a superior drug development strategy, as it has the potential to reduce costs and shorten the duration of clinical trial cycles (Riva et al., 2020; Wu C. et al., 2020). Network pharmacology identifies pertinent disease targets for the treatment of Parkinson’s disease through drug-target networks and experimentally validates the analyzed pathways and mechanisms through enrichment analysis. (Gao et al., 2024; Sasikumar et al., 2024). The development of drug-target networks constitutes a crucial methodology in computational drug repurposing.

**FIGURE 1:**
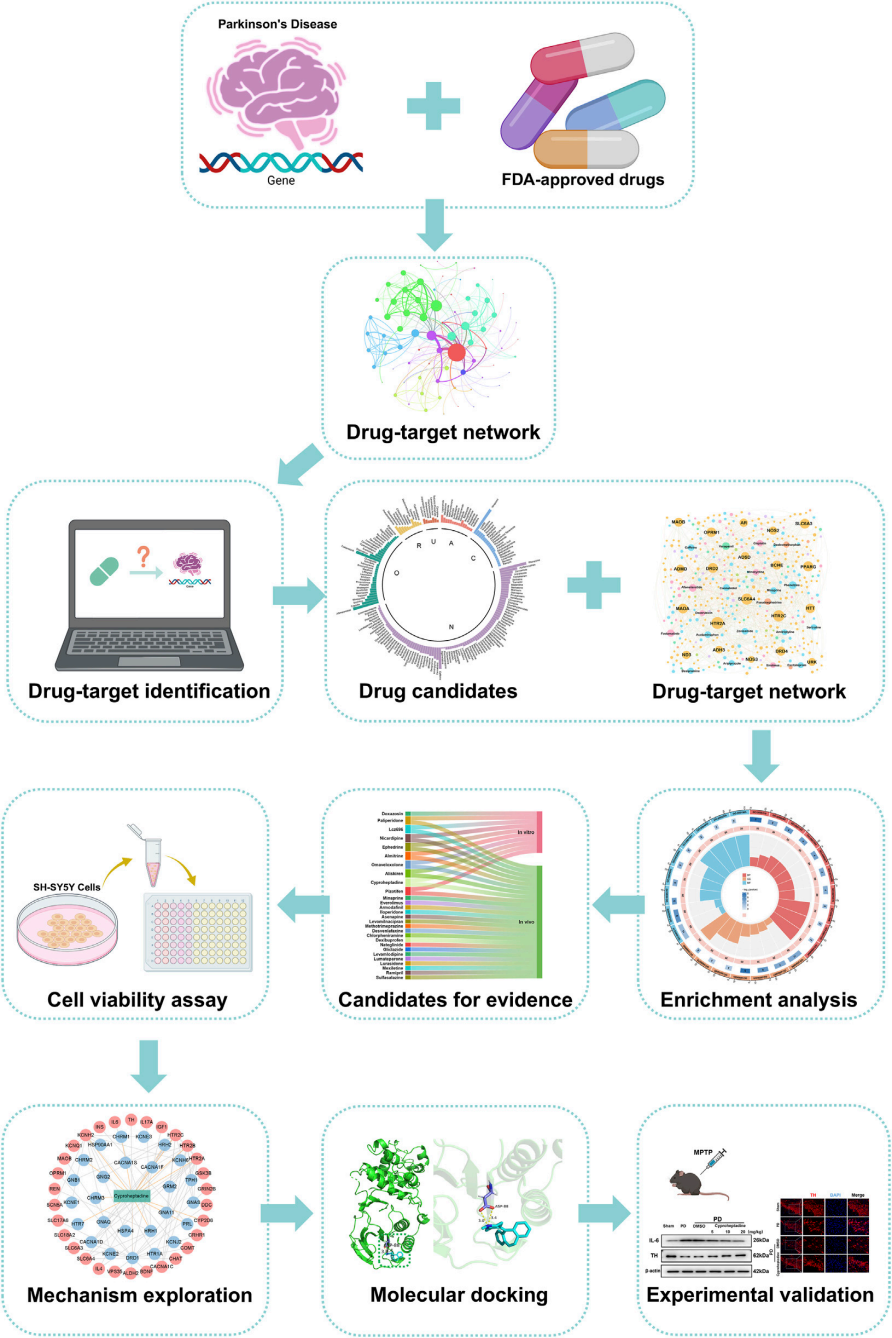
Flowchart of experimental design.

Traditional drug development is time-consuming and costly, driving drug repurposing as an innovative strategy. It uses computer-aided design to identify potential therapeutics from existing drugs, shortening timelines and reducing costs. In this study, we employ a drug-target network-based approach to screen for PD treatments, analyzing drug-disease target interactions to enhance screening accuracy. We focus on repurposing FDA-approved drugs due to their established safety profiles, which reduce risks and development time compared to new drug development. Our methodological framework is outlined in Figure 1. We screen approved drugs from DrugBank and other databases for potential PD treatment. Using the NetInfer database to predict drug targets, we assume drugs with targets overlapping with PD targets may be effective. This approach allowed us to narrow down from 2,453 FDA-approved drugs to 176 potential candidates. Following literature review and *in vitro* experiments, we found Omaveloxolone and Cyproheptadine exhibited neuroprotective effects in PD cell models. Further *in vivo* studies revealed Cyproheptadine inhibits IL-6 expression and prevents TH downregulation via the MAPK/NFκB pathway, while Omaveloxolone likely reduces oxidative stress damage and protects dopaminergic neurons by activating the Keap1-Nrf2/ARE pathway, thereby confirming the effectiveness of our computational drug repurposing approach.

## 2 Materials and methods

### 2.1 Collection of Parkinson’s disease genes

The collection of Parkinson’s disease genes is primarily from six authoritative databases, including the ClinGen database (https://www.clinicalgenome.org/), the ClinVar database (https://www.ncbi.nlm.nih.gov/clinvar/), the DisGeNET database (https://www.disgenet.org/), the GeneCards database (https://www.genecards.org/), the OMIM database (https://www.omim.org/) and the Phenopedia database (https://phgkb.cdc.gov/PHGKB/startPagePhenoPedia.action) (Huang, 2023). The search term we entered on these databases was “Parkinson’s disease”. For the ClinVar database, we only keep genes with the number of stars greater than or equal to two. For the DisGeNET database, our screening criteria were genes with DisGENET score ≥0.1 and evidence index (EI) >0. For the GeneCards database, we retained genes with a SCORE greater than 25. For the Phenopedia database, we retained the genes that have been reported to be associated with Parkinson’s disease. Parkinson’s disease genes from these six database searches were combined and de-emphasized to obtain 1,086 genes (Supplementary Table S1).

### 2.2 Integration of the human protein-protein interactome

All genes are arranged according to Betweenness. We first selected 272 genes (top 25%) of Parkinson’s disease genes into THE STRING database (https://cn.string-db.org/) for human protein-protein interactions (PPIs) network analysis (Huang, 2023). The raw data were exported and constructed using Cytoscape (version 3.9.1).

### 2.3 Construction of drug-target network

We first collected Parkinson’s disease gene-related drugs through six authoritative drug databases, including the BindingDB database (https://www.bindingdb.org/bind/index.jsp), the ChEMBL database (https://www.ebi.ac.uk/chembl/), the DrugBank database (https://go.drugbank.com/), the IUPHARBPS Guide to PHARMACOLOGY database (https://www.guidetopharmacology.org/), the PharmGKB database (https://www.pharmgkb.org/), and the Therapeutic Target Database (https://db.idrblab.net/ttd/) (Fang et al., 2021). The search term we entered on these databases was “Parkinson’s disease”. Then we searched and got FDA-approved 2,453 drugs (Supplementary Table S2). After we removed the genes with no drug targets, we ended up assembling 6,164 Drug–Target Interactions (DTIs), made up of 526 Parkinson’s disease genes as well as 2,453 FDA-approved drug interactions (Supplementary Table S3). The Drug-target network were constructed by Gephi (version 0.9.7).

### 2.4 Identification of potential drug candidates for Parkinson’s disease

In this study, we used the NetInfer database (http://lmmd.ecust.edu.cn/netinfer/) for web-based online drug target prediction (Alpha (α):0.4, Beta (β):0.2, Gamma (γ): 0.5, Delta (δ):20, Epsilon (ε):4, Number of resource-diffusion processes (k):2, Number of predictions for each compound:100), and we hypothesized that if a drug’s primary predicted target overlaps with a Parkinson’s disease target, then the drug is likely to hold promise for treating Parkinson’s disease (Wu Z. et al., 2020). In the end, we screened 176 drugs that possess the potential to treat Parkinson’s disease (Supplementary Table S4).

### 2.5 Reagents

Paliperidone, Lcz696, Nicardipine, Almitrine, Omaveloxolone, Aliskiren, Cyproheptadine and Pizotifen were purchased from Selleck (Shanghai, China), dissolved in 2% dimethyl sulfoxide (DMSO) and diluted with 0.9% saline to a stock solution. Rabbit anti-IL-6 was purchased from Abcam (1:1,000, ab259341). Rabbit anti-TH was purchased from Servicebio (GB15182-100, Wuhan, China). Rabbit anti-Nrf2 was purchased from Affinity Biosciences (1:1,000, AF0639, Changzhou, China). Rabbit anti-β-actin (1:1,000, #4970), Rabbit anti-P-ERK (1:1,000, #4370), Rabbit anti-ERK (1:1,000, #4695), Rabbit anti- P-JNK (1:1,000, #4668), Rabbit anti- JNK (1:1,000, #9252) and Rabbit anti- KEAP1 (1:1,000, #8047) was purchased from Cell Signaling Biotechnology. Rabbit anti-P-P38 (1:1,000, AP0526), Rabbit anti-NQO-1 (1:1,000, A23486) and Rabbit anti-HO-1 (1:1,000, A21911) was purchased from Abclonal (Wuhan, China). Rabbit anti-P-P38 (1:1,000, AP0526), Rabbit anti-NQO-1 (1:1,000, A23486) and Rabbit anti-HO-1 (1:1,000, A21911) was purchased from Abclonal (Wuhan, China). Rabbit anti-P38 (1:1,000, AF1111), Rabbit anti-P-P65 (1:1,000, AF5875) and Rabbit anti- P65 (1:1,000, AF1234) was purchased from Beyotime (Shanghai, China). ECL chemiluminescence system was obtained from Thermo Company (Rockford, IL, United States).

### 2.6 Animals

Adult male C57BL/6J (B6) mice (8–10 weeks, 25–30 g) were provided by Yangzhou University Comparative Medical College. The animals were housed in a standard SPF-grade animal house at a temperature (22°C ± 2°C) and 60% humidity, with a regular 12 h light/12 h dark cycle and free access to water and food. The experimental protocol and all experimental procedures were approved by the Animal Ethics Committee of Yangzhou University School of Medicine.

### 2.7 Grouping and drug administration

A total of 132 mice were used in our study, and the specific subgroups and the number used in each group can be categorized into the following five groups: (a) Sham group (Sham, n = 30), (b) Parkinson’s disease mice (PD, n = 30), (c) DMSO-treated mice with Parkinson’s disease group (PD + DMSO, n = 30), (d) Cyproheptadine-treated mice with Parkinson’s disease group (PD + Cyproheptadine, n = 21), (e) Omaveloxolone-treated mice with Parkinson’s disease group (PD + Omaveloxolone, n = 21). For Western blot, ELISA, and immunofluorescence analyses, three mice were used per group. The open-field test included six mice per group. Cyproheptadine (5 mg/kg, 10 mg/kg, 20 mg/kg) and Omaveloxolone (5 mg/kg, 10 mg/kg, 20 mg/kg) were injected intraperitoneally (i.p.) once a day starting on the fifth day of induction of the PD mouse model. Equal volumes of saline were injected intraperitoneally in the sham group. For the PD + DMSO group, an equal volume of vehicle (2% DMSO in normal saline) was injected intraperitoneally.

### 2.8 MPTP-induced model of Parkinson’s disease

MPTP was purchased from sigma (MO, United States). The MPTP-induced PD model is widely used to mimic dopaminergic neurodegeneration through systemic inhibition of mitochondrial complex I, causing rapid and selective loss of TH-positive neurons in the substantia nigra (Li et al., 2025). Remove the prepared stock solution of MPTP stored at −20°C and dilute to the appropriate concentration using sterile PBS. Mice were uniformly injected intraperitoneally with diluted MPTP solution (30 mg/kg) once a day for 1 week (Boonpraman et al., 2023). Successful modeling was marked by a stiff tail, involuntary tremors in the body, and reduced activity in the mice.

### 2.9 Open field test

Behavioral testing began on the second day of PD modeling, which was assessed for an independent researcher who was unaware of the experimental group. The open field test is designed to evaluate autonomous behavior as well as exploratory behavior in mice and is widely used in Parkinson’s disease models (Yan et al., 2023). The experimental animals were placed in the central area of the open field, and the mice were allowed to move freely, and the activity of the mice in the open field for 5 min was recorded. After the experimental testing is completed and each mouse is removed, the open field should be thoroughly cleaned to avoid affecting the next results.

### 2.10 Western blot analysis

Tissues from the mouse substantia nigra region were collected and added to RIPA lysis buffer (Millipore, Billerica, MA, United States) with 1% protease inhibitor dissolved on ice (Guo et al., 2021). The BCA protein assay kit (Thermo-Fisher Scientific, MA, United States) was standardized for protein quantification. Equal concentrations of proteins were applied to a 12.5% sodium dodecyl sulfate-polyacrylamide gel for electrophoresis (SDS-PAGE) and then electro transferred to PVDF membranes (Millipore). The membranes were blocked in Tris-buffered saline-Tween (TBST) containing 5% skimmed milk for 2 h, followed by an overnight incubation with primary antibodies at 4°C. The next day, the secondary antibody was incubated at room temperature after three TBST washes. And scanned using an ECL chemiluminescence system (Thermo Company, West Chester, PA, United States). Measure and quantitate the intensity of protein bands using ImageJ image analysis software.

### 2.11 Immunofluorescence staining

Place the frozen sections stored at −80°C in room temperature for 15 min. Fixed with 4% paraformaldehyde for 15 min, Then fixed with 4% paraformaldehyde for 15 min, and then washed in PBS three times for 5 min each time. Add 0.25% PBST solution for membrane rupture for 25 min and washing with PBS three times. These sections were blocked for 2 h using 2% BSA, followed by an overnight incubation with primary antibodies at 4°C. After washing with PBS three times, Incubate the secondary antibody (Invitrogen, 1:500) for 2 h at room temperature in the dark, and then washed with PBS three times for 15 min. Immunofluorescence results were obtained using a fluorescence microscope after staining with DAPI solution for 5 min. Images were obtained using a fluorescence microscope (Olympus X73, Fukasawa, Japan). Average fluorescence intensity statistics were performed using ImageJ, and graphs were plotted and statistically analyzed using GraphPad Prism 8.0.

### 2.12 Cell proliferation assay

SH-SY5Y cells were purchased from Pricela (Wuhan, China). Cell proliferation assay was measured by CCK8 kit (Beyotime, China) according to the manufacturer’s instructions. SH-SY5Y cells were seeded in a 96-well plate, and then diluted MPP^+^ (Sigma, United States) (5 μM) was added to DMEM to induce Parkinson’s disease *in vitro* model for 72 h (Yao et al., 2023). After the induction is completed, the medium is changed and different concentrations of drugs are added to stimulate and the cell proliferation assay is performed for 24 h.

### 2.13 Measurement of IL-6, SOD activity levels

Brain tissue from the substantia nigra area of the PD mouse model was collected and homogenized. Enzyme-linked immunosorbent assay kits of IL-6 and SOD were purchased from Beyotime (Wuhan, China). Abundance levels of IL-6 and SOD were measured using commercial assay kits according to the manufacturer’s instructions.

### 2.14 Molecular docking

We downloaded the 3D structures of P38 (PDB DOI: https://doi.org/10.2210/pdb6SFO/pdb) and Kelch structural domain of KEAP1 (PDB DOI: https://doi.org/10.2210/pdb6rog/pdb) from the Protein Data Bank database (http://www.rcsb.org/) (Xu et al., 1997; Bueno-Carrasco et al., 2022). The 3D structures of Omaveloxolone and Cyproheptadinewere obtained from the PubChem database (https://pubchem.ncbi.nlm.nih.gov/). The prepared target protein was subjected to hydrogenation, charge calculation, and addition of atom types using AutoDock software to obtain a ligand-free protein, imported small molecule drugs, set the center coordinates and size of the gridbox, docked the protein and the small molecule drug, and the docked file was imported into Pymol software for visual mapping analysis. It is generally believed that the lower the binding energy, the better the docking effect.

### 2.15 Statistical analysis of the data

All data are expressed as mean ± SEM of at least three independent experiments. All experimental data were analyzed by observers who were unaware of the experimental protocol. All relevant data were tested for normal distribution by SPSS 25.0 software. Correlation statistics were analyzed and plotted using GraphPad Prism 8.0 and ImageJ, and t-tests were performed using two-group means. Comparisons between multiple groups were performed using one-way analysis of variance (ANOVA) followed by Tukey’s multiple comparison test, with P < 0.05 being considered statistically significant.

## 3 Results

### 3.1 PPI network and enrichment analysis of Parkinson’s disease genes

**FIGURE 2:**
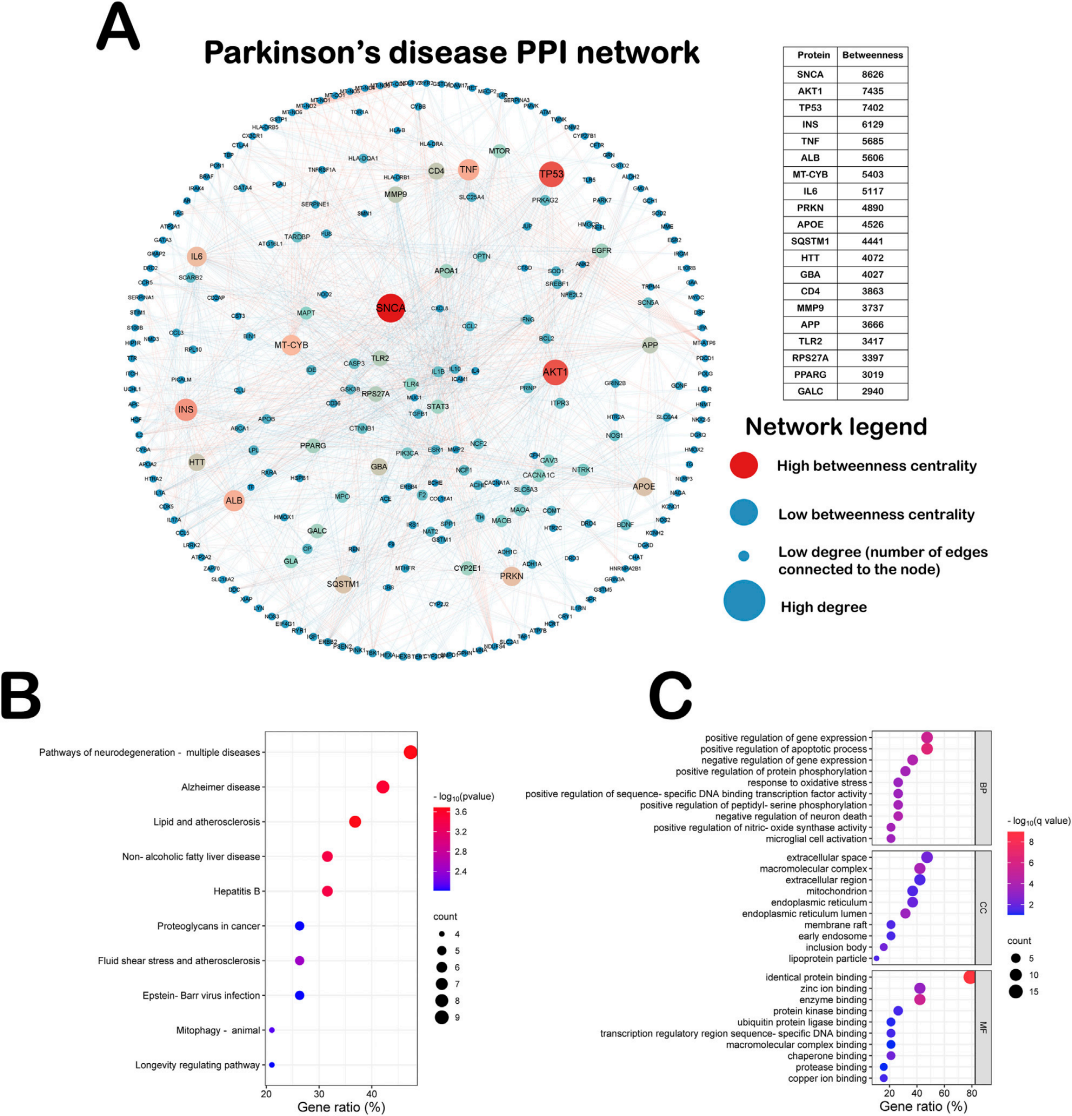
PPI network and enrichment analysis of Parkinson’s disease genes. **(A)** This PPI network includes 272 Parkinson’s genes and 1,518 edges. **(B)** KEGG pathway analyses on the top twenty ranked Parkinson’s disease genes. **(C)** GO pathway analyses on the top twenty ranked Parkinson’s disease genes.

We integrated the PPI network of Parkinson’s disease through six authoritative libraries, which was designed to explore the effects of different causative genes of Parkinson’s disease on the interaction network. As shown in Figure 2A, this PPI network includes 272 Parkinson’s genes and 1,518 edges. The top 20 Parkinson’s disease genes according to the degree of Betweenness centrality are SNCA, AKT1, TP53, INS, TNF, ALB, MT-CYB, IL-6, PRKN, APOE, SQSTM1, HTT, GBA, CD4, MMP9, APP, TLR2, RPS27A, PPARG and GALC. All of these genes play an important role in Parkinson’s disease. Mutations in the autosomal dominant gene SNCA and the autosomal recessive gene PRKN can cause hereditary Parkinson’s disease (Cherian et al., 2023). Therefore, in order to explore the involvement of these genes in biological functions as well as potential pathways, we performed KEGG pathway analyses and Gene Ontology (GO) on the top twenty ranked Parkinson’s disease genes (Figures 2B,C). As shown in Figure 2B, the most significantly enriched pathway in the KEGG pathway analysis was the neurodegenerative disease pathway, demonstrating the accuracy of this PPI network. As shown in Figure 2C, Biological Processes (BP) in GO analysis suggest that these causative genes mainly affect the apoptotic process, oxidative stress response, Death of neurons and activation of microglia in Parkinson’s disease (Emerit et al., 2004). Analysis of the Cellular Components (CC) of these genes showed predominantly in the endoplasmic reticulum and mitochondria. Molecular Functions (MF) are primarily involved in enzyme binding, including protein kinases and ubiquitination ligases. Evidence suggests that oxidative stress arises mainly as a result of redox imbalance of reactive oxygen species (ROS) and reactive nitrogen species (RNS). The production of oxidative stress activates the activation of the neuronal apoptotic program, leading to neuronal death. It also promotes the activation of microglia, which secrete inflammatory factors and further exacerbate the death of dopaminergic neurons (Emerit et al., 2004).

### 3.2 Drug-target network analysis of Parkinson’s disease

**FIGURE 3:**
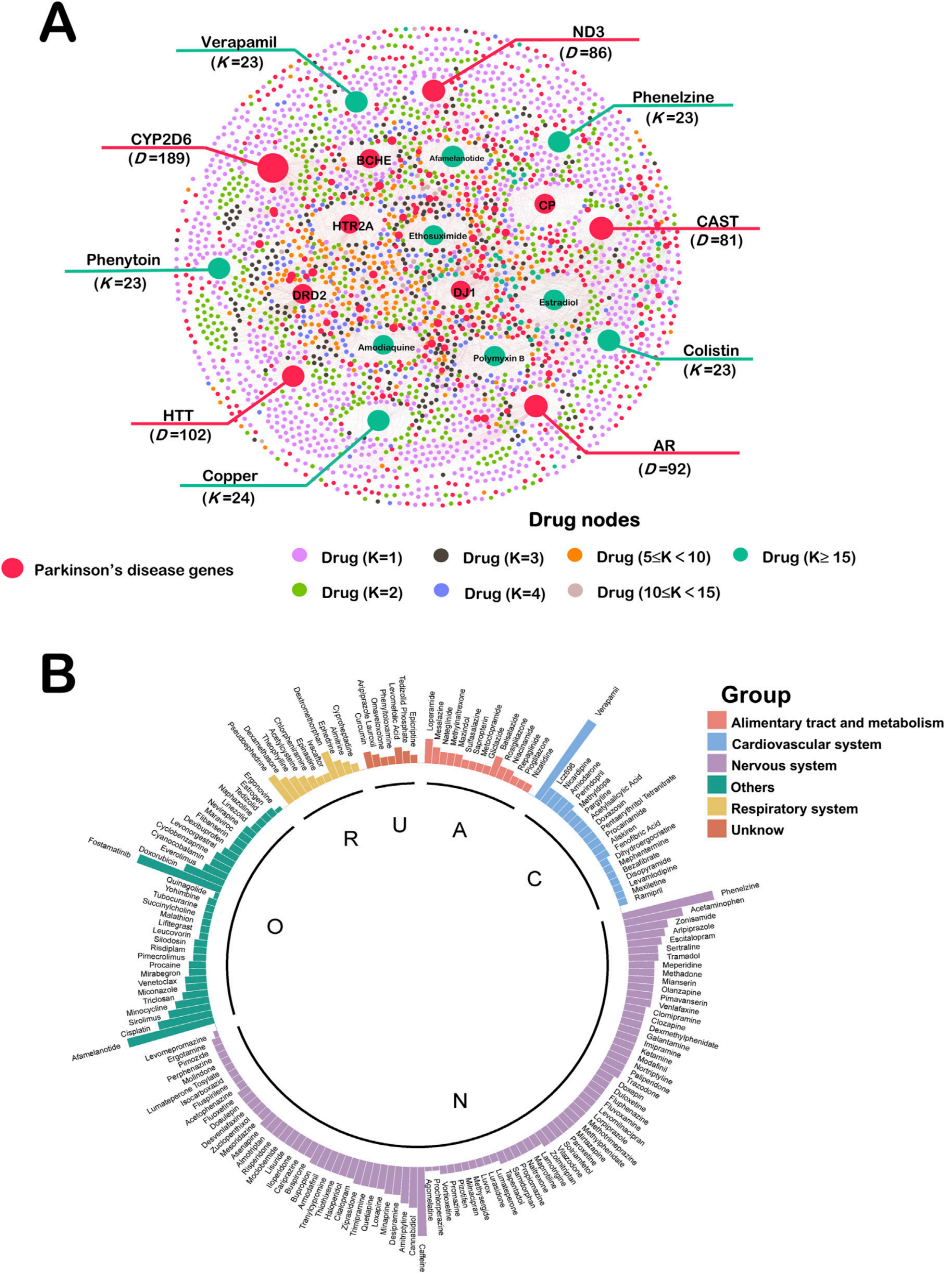
**(A)** Drug-target network of Parkinson’s disease. This network ended up assembling 6,164 DTIs, made up of 526 Parkinson’s disease genes as well as 2453 FDA-approved drug interactions. These top 10 disease genes and therapeutic drugs are labeled. Drug nodes were classified by the degree (K). The sizes of node and label are proportional to degree. **(B)** Computational Drug Repurposing for Parkinson’s Disease. There are 176 drugs calculated to be repurposed as Parkinson’s disease candidates. These drugs were categorized by ATC into six major groups. A: alimentary tract and metabolism (n = 15); C: cardiovascular system (n = 20); N: nervous system (n = 89); O: other (n = 34); R: respiratory system (n = 11); U: unknow (n = 7).

We constructed a network of Drug-target network for Parkinson’s disease (Figure 3A). This network ended up assembling 6,164 Drug–Target Interactions (DTIs), made up of 526 Parkinson’s disease genes as well as 2453 FDA-approved drug interactions. These top 10 disease genes and therapeutic drugs are labeled in the Figure 3A. We use “D” to represent the number of drugs attached to each gene. These genes are CYP2D6 (D = 189), HTT (D = 102), AR (D = 92), ND3 (D = 86), CAST (D = 81), HTR2A (D = 80), CP (D = 78), DJ1 (D = 76), DRD2 (D = 74) and BCHE (D = 66). There’s evidence to suggest that ND3 gene polymorphisms have been associated with a protective effect against PD (Castañeda et al., 2022). Genetic variant of HTR2A associates with risk of impulse control and repetitive behaviors in Parkinson’s disease (Lee et al., 2012). We use “K” to represent the number of genes attached to each drug. The top ten drugs in the Drug Target Network were Copper (K = 24), Colistin (K = 23), Phenelzine (K = 23), Phenytoin (K = 23), Verapamil (K = 23), Estradiol (K = 22), Polymyxin B (K = 22), Afamelanotide (K = 21), Amodiaquine (K = 21) and Ethosuximide (K = 21). These drugs have great potential in the treatment of PD and some of them have been reported in the literature.

### 3.3 Computational drug repurposing for Parkinson’s disease

In this study, we screened 176 PD drug candidates through the NetInfer database. These drugs were categorized by Anatomical Therapeutic Chemical (ATC) into six major groups including Alimentary tract and metabolism (n = 15), Cardiovascular system (n = 20), Nervous system (n = 89), Other (n = 34), Respiratory system (n = 11) and Unknow (n = 7) (Figure 3B). The height of each drug is related to the number of drug targets it enriches. From our results, 50.6% of the drug candidates belonged to the nervous system, which is consistent with PD being a neurological disorder. Drug candidates for the alimentary tract and metabolism, cardiovascular, and respiratory systems have also previously been shown to work in the nervous system (Kittaka et al., 1997; Bavarsad Shahripour et al., 2014; Saad et al., 2020).

### 3.4 Network analysis of repurposed Parkinson’s disease drug-target

**FIGURE 4:**
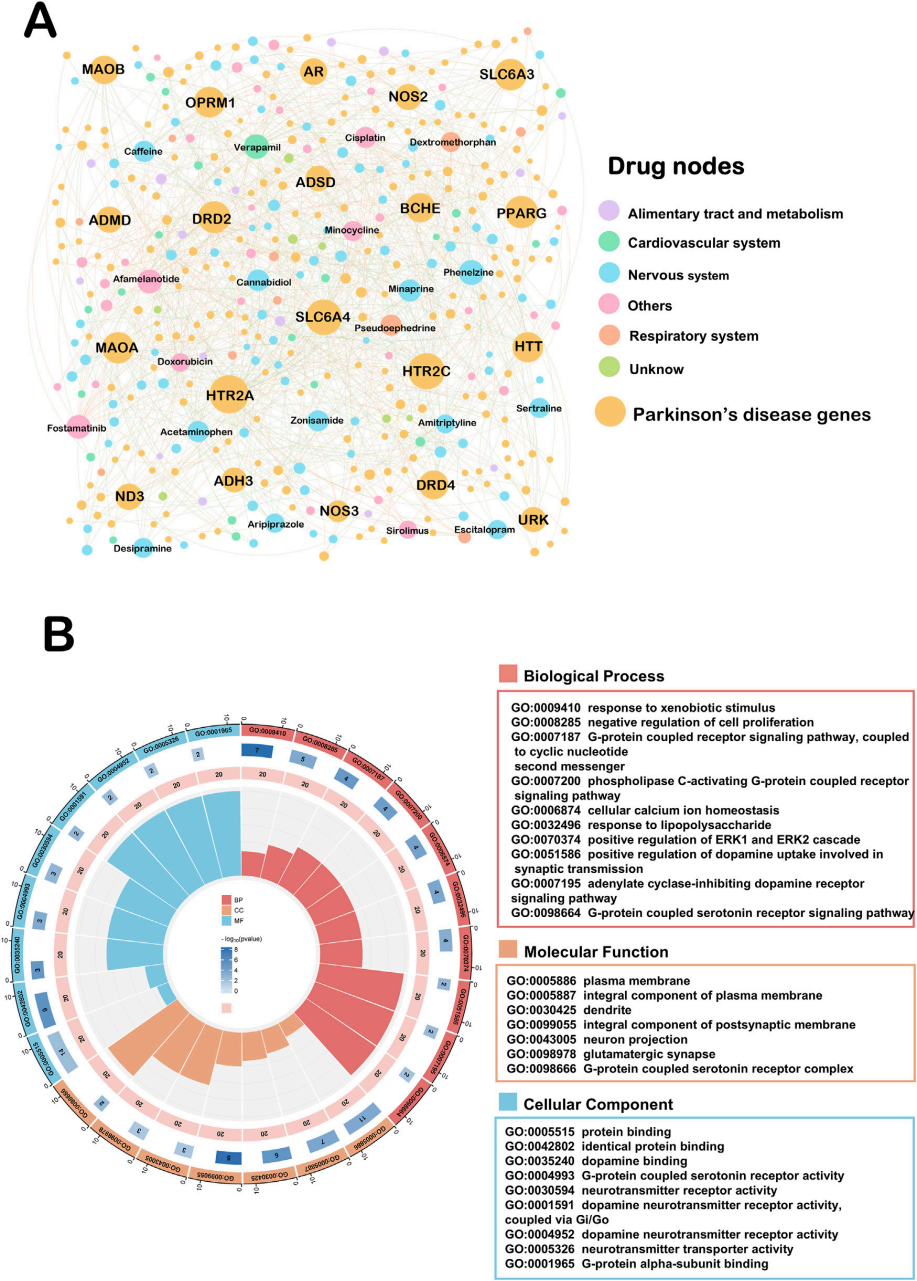
Drug-target network of repurposed for Parkinson’s disease. **(A)** This network ended up assembling 695 DTIs, made up of 225 Parkinson’s disease genes as well as 176 drug candidates interactions. Drug nodes were classified by the degree (K). The sizes of node and label are proportional to degree. **(B)** GO pathway analyses on the top twenty ranked the Drug-target network.

In order to visualize the relationship between our screened drug candidates and disease targets, we again mapped the repurposed Drug-target network (Figure 4A). This network ended up assembling 695 DTIs, made up of 225 Parkinson’s disease genes as well as 176 drug candidates interactions. The top five drugs in this network based on connectivity were verapamil (K = 22), Fostamatinib (K = 21), Afamelanotide (K = 20), Phenelzine (K = 20) and Caffeine (K = 17). A number of these drugs have been reported to exert neuroprotective effects in the nervous system (Ismael et al., 2022; Stanislaus et al., 2023). To further explore how these drugs are involved in the generation of biological functions, we again performed GO analysis of the top 20 Parkinson’s disease genes (Figure 4B). Regarding BP analysis, we can find ten biological processes enriched to three G-protein coupled receptor signaling pathway related. There is evidence that G protein-coupled receptors, as molecules involved in signaling across cell membranes, are involved in the receptors that transmit the action of dopamine in the brain, known as dopamine receptors (Gurevich et al., 2016). Regarding MF analysis, which is enriched to the integral component of postsynaptic membrane and glutamatergic synapse. Evidence suggests that Parkinson’s disease glutamatergic prominence is important for posterior signaling, function, and plasticity (Moraes et al., 2021). Reuptake of glutamate in the striatum to ameliorate motor dysfunction in PD model rats (Feng et al., 2021). For cellular components, G-protein-coupled serotonin receptor activity and G-protein alpha-subunit binding were enriched. It is worth mentioning that BP, MF and CC analyses were simultaneously enriched for the G-protein coupled receptor signaling pathway.

### 3.5 Parkinson’s disease therapeutic agents with therapeutic potential and *in vitro* experimental validation

**FIGURE 5:**
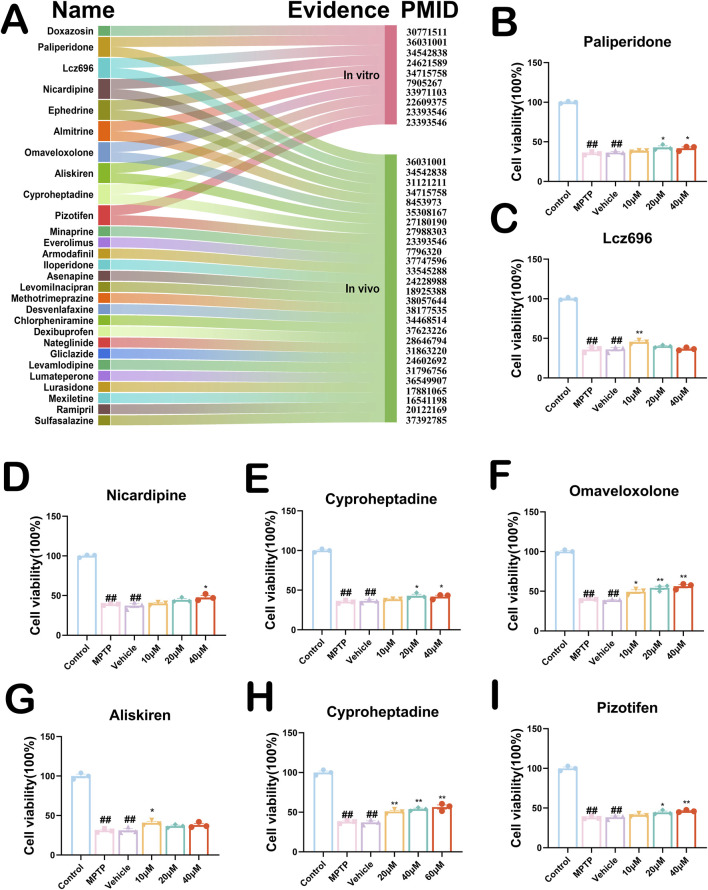
**(A)** Twenty-eight PD drug candidates with neuroprotective effects. PMID stands for Specific Literature Evidence of Prior Neuroprotective Effects of the Drug. Evidence is categorized into *in vivo* and *in vitro* representations. Nine drug candidates have been shown to have neurocellular protective effects *in vivo* and *in vitro*. **(B–I)** CCK8 experiments validate effects of drug candidates on SH-SY5Y cells. Above are the results of testing the activity of eight drugs on MPTP-induced SH-SY5Y cells. All data are presented as mean ± SEM, n = 3. The eight drugs in order were Paliperidone, Lcz696, Nicardipine, Almitrine, Omaveloxolone, Aliskiren, Cyproheptadine and Pizotifen. ##*P* < 0.01 compared with the control group. *P < 0.05 and **P < 0.01 compared with the MPTP group.

To better optimize the accuracy of this computational drug repurposing scheme of ours, we systematically searched the literature for the 176 PD drug candidates that we have screened. We found seventy (39.8%) drugs with proven anti-PD effects *in vivo*, *in vitro*, or clinically. Eighty-sixth (48.9%) drugs had no evidence of an anti-PD effect, and the rest of the remaining existed with evidence of clear harmful effects on PD. To explore the anti-PD potential of these drugs, we identified twenty-eight drugs with proven neurocytoprotective effects by searching the literature on these drugs (Figure 5A). To further improve the efficiency as well as accuracy of our drug repurposing, we selected only eight drug candidates that have been shown to have neuroprotective effects *in vivo* and *in vitro* (Figures 5B–I). Ephedrine could not be detected because it was not available. The results of our CCK8 experiments suggested that both Omaveloxolone (20 μM and 40 μM) and Cyproheptadine (20 μM, 40 μM and 60 μM) significantly increased the cell proliferative activity of SH-SY5Y cells with increasing drug concentrations (Figures 5F, H, *P* < 0.01). Lcz696 significantly increased the cell proliferative activity of SH-SY5Y only at low concentrations (10 μM) (Figure 5C, 38.9639–51.6559 vs. 26.8574–45.1034, *P* = 0.0043). Aliskiren and Almitrine increased the cell proliferative activity of SH-SY5Y cells only at low concentrations (10 μM), which were statistically different (Figures 5E,G, *P* < 0.05). Paliperidone and Pizotifen did not increase SH-SY5Y cell proliferative activity at low concentrations (10 μM), but were able to increase SH-SY5Y cell proliferative activity at medium (20 μM) and high concentrations (40 μM) (Figures 5B,I, *P* < 0.05). Nicardipine increases the cell proliferative activity of SH-SY5Y cells only at high dose concentrations (40 μM) (Figure 5D, 37.8818–57.7898 vs. 35.6859–42.5339, *P* = 0.0169). These drugs showed some neuroprotective effects in the MPTP-treated SH-SY5Y cell model, especially Omaveloxolone and Cyproheptadine.

### 3.6 Mechanistic exploration of cyproheptadine against PD

**FIGURE 6:**
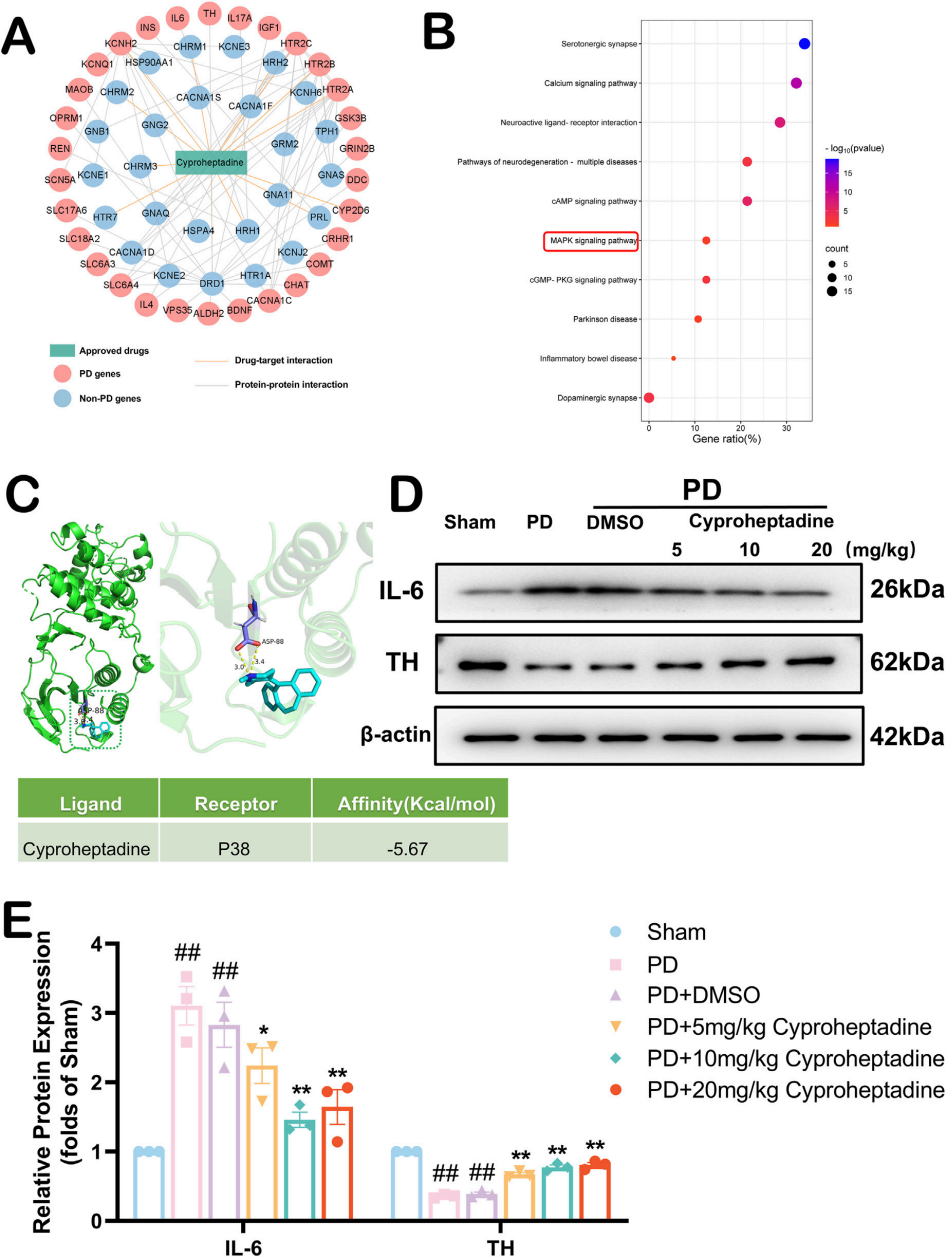
Exploration of the anti-PD mechanism of cyproheptadine. **(A)** The drug-target and protein-protein interaction network of cyproheptadine. **(B)** KEGG pathway analyses. **(C)** Schematic representation of molecular docking of cyproheptadine with P38 and Molecular Docking Energy Scale. **(D)** The representative images show the levels of IL-6, TH and β-actin by Western blotting. **(E)** Quantitative analysis showed that the expression levels of IL-6 and TH. All data are presented as mean ± SEM, n = 3. The PD + Cyproheptadine group showed decreased expression of IL-6 and elevated expression of TH compared to the PD group. ##P < 0.01 compared with the control group. *P < 0.05 and **P < 0.01 compared with the MPTP group.

To explore the anti-PD neuroprotective mechanism of action of Cyproheptadine, we constructed a Drug-target network for this drug (Figure 6A). This drug target network demonstrates that Cyproheptadine connects to 5 PD genes and 61 PPI partners (e.g., IL-6 and TH). IL-6 serves as a marker of neuroinflammation in PD, and drugs targeting this target have good potential in anti-PD (Liu et al., 2022). Increased TH expression in the substantia nigra area represents an increase in the number of dopaminergic neurons in this region (Jiao et al., 2024). To further explore the potential mechanisms by which Cyproheptadine protects against PD, we subjected 56 genes associated with PD to KEGG pathway analysis (Figures 6A,B). The results of KEGG pathway analysis suggest that the MAPK signaling pathway may be the signaling pathway for Cyproheptadine against PD. So we explored whether Cyproheptadine binds to P38 by computer-simulated molecular docking. In Figure 6C, We found that Cyproheptadine is capable of binding to P38 by computer-simulation methods. The docking scores for the combination is −5.67 kcal/mol respectively. To better demonstrate the effectiveness of Cyproheptadine, we constructed a PD mouse model. We examined the protein expression of IL-6 and TH in the mouse substantia nigra area by Western blot (Figure 6D). We used three different concentrations (5 mg/kg, 10 mg/kg, 20 mg/kg) of Cyproheptadine treatments in the PD mouse model. In Figure 6E, The PD + Cyproheptadine group showed decreased expression of IL-6 and prevented the downregulation of TH compared to the PD group (*P* < 0.05). Cyproheptadine at 10 mg/kg and 20 mg/kg significantly reduced IL-6 expression in PD mice (*P* < 0.01), but the 20 mg/kg dosage may have resulted in a slight upregulation of IL-6 relative to 10 mg/kg due to high drug concentrations. As the drug concentration of Cyproheptadine was elevated, the expression of TH was significantly increased (*P* < 0.01). Taken together, we believe that the concentration of 10 mg/kg is the optimal concentration for Cyproheptadine to exert its anti-PD effects in the PD mouse model.

### 3.7 Cyproheptadine increases dopaminergic neuron expression to ameliorate neurologic deficits in PD via the MAPK/NFκB signaling pathway

**FIGURE 7:**
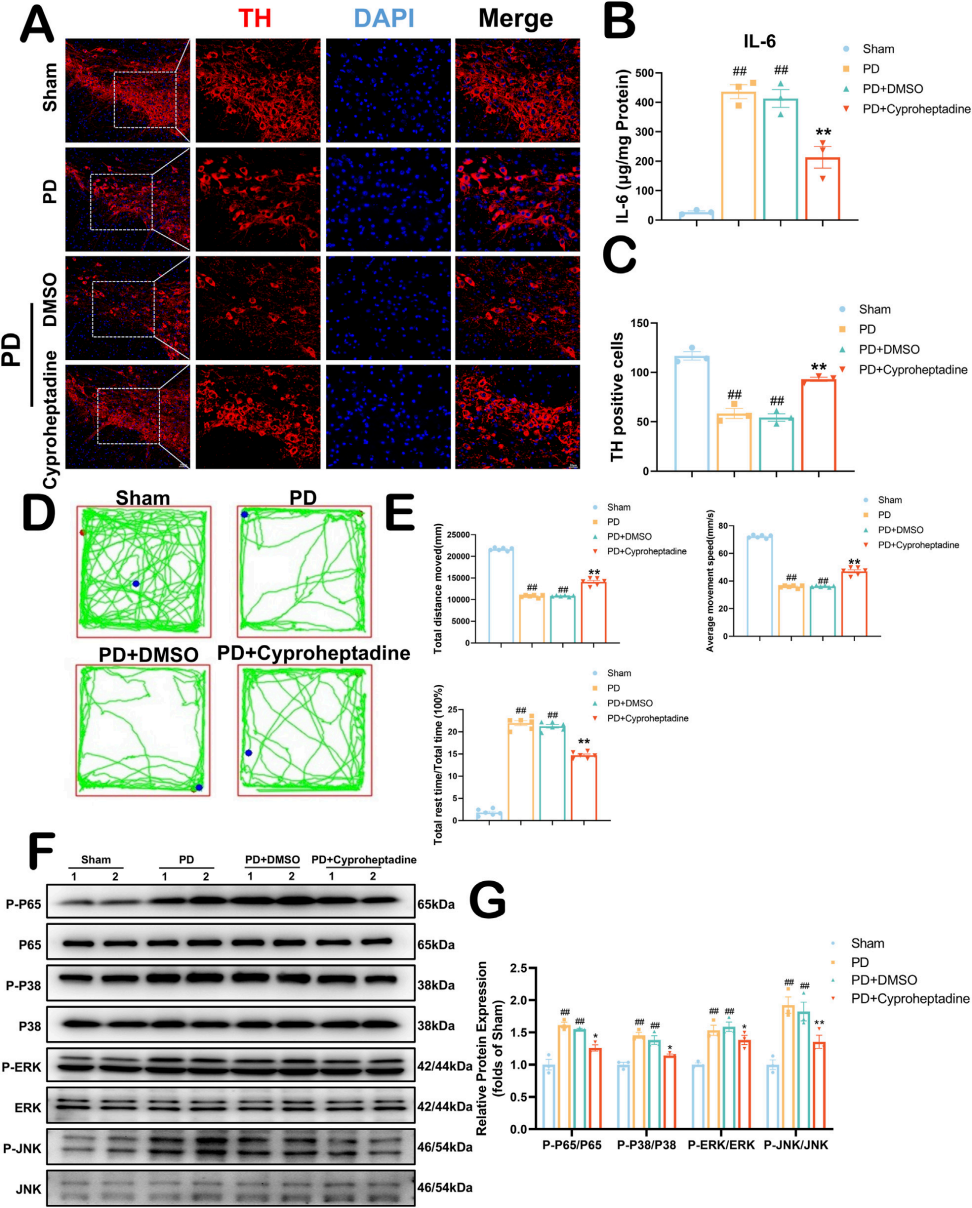
Cyproheptadine increases dopaminergic neuron expression to ameliorate neurologic deficits in PD via the MAPK/NFκB signaling pathway. **(A)** Survival of dopaminergic neurons detected by TH fluorescence staining of the substantia nigra area. **(B)** Expression of IL-6 in the substantia nigra area by ELISA. All data are presented as mean ± SEM, n = 3. Cyproheptadine significantly reduced the expression of IL-6 in mice in the PD group. **(C)** Cyproheptadine significantly increases the survival of dopaminergic neurons in the substantia nigra area of PD mice. All data are presented as mean ± SEM, n = 3. **(D)** Neurological behavior of mice detected by Open field test. **(E)** The PD + Cyproheptadine group was significantly improved compared to the PD group in terms of total distance moved and average movement speed, and significantly less in terms of the proportion of rest time. All data are presented as mean ± SEM, n = 6. **(F)** The representative images show the levels of P-P65, P-P38, P-ERK and P-JNK by Western blotting. P65, P38, ERK and JNK were used as a loading control. **(G)** Quantitative analysis of P-P65/P65, P-P38/P38, P-ERK/ERK and P-JNK/JNK ratio. All data are presented as mean ± SEM, n = 3. ##P < 0.01 compared with the control group. **P < 0.01 compared with the MPTP group.

We examined the expression of dopaminergic neurons in the mouse substantia nigra area by immunofluorescence (Figure 7A). *In vivo*, we used 10 mg/kg of Cyproheptadine to treat PD mice. There was no statistically significant difference in TH expression between the PD and PD + DMSO groups (36.63–80.03 vs. 38.37–70.29, *P* = 0.9617). The PD + Cyproheptadine group significantly prevented the downregulation of TH and decreased dopaminergic neuron loss compared to the PD group (Figure 7C, 93.69–104.97 vs. 42.41–62.93, *P* = 0.0028). In Figure 7B, We examined the expression of IL-6 in the mouse substantia nigra area by ELISA kit. PD + Cyproheptadine group significantly reduced IL-6 expression levels in PD mice (53.965–372.565 vs. 334.1995–537.9595, *P* = 0.0068). We used the absentee field test to assess neurological deficits in Parkinson’s model mice, including aspects of autonomous behavior and exploratory behavior (Boonpraman et al., 2023) (Figure 7D). In Figure 7E, There were no differences between the PD and PD + DMSO groups in terms of total distance moved, average movement speed, and proportion of rest time (*P* > 0.05). The PD + Cyproheptadine group was significantly improved compared to the PD group in terms of total distance moved and average movement speed, and significantly less in terms of the proportion of rest time (*P* < 0.01). In Figure 6B, the results of KEGG pathway analysis suggest that the MAPK signaling pathway may be the signaling pathway for Cyproheptadine against PD. Thus, we verified that Cyproheptadine acts through the MAPK/NFκB signaling pathway against PD (Figure 7F). We found that the PD + Cyproheptadine group was able to significantly decrease the levels of P-P65, P-P38, P-ERK and P-JNK compared to the PD + DMSO group (Figure 7G, P < 0.01). These results are suggestive of the ability of Cyproheptadine to reduce neuroinflammation, increase dopaminergic neuron expression, and attenuate neurological deficits in PD mice via the MAPK/NFκB signaling pathway.

### 3.8 Mechanistic exploration of Omaveloxolone against PD

**FIGURE 8:**
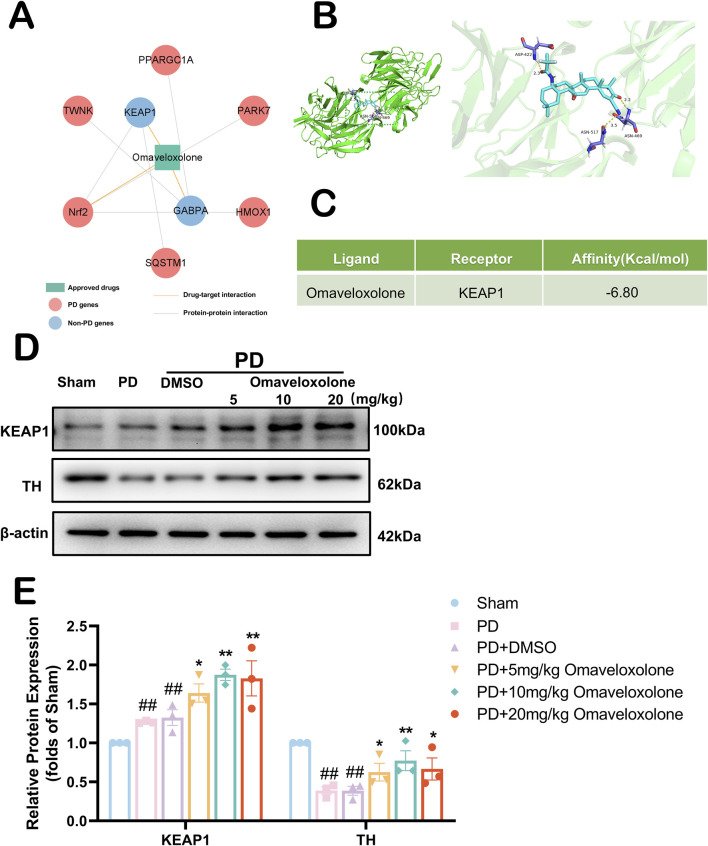
Exploration of the anti-PD mechanism of Omaveloxolone. **(A)** The drug-target and protein-protein interaction network of Omaveloxolone. **(B)** Schematic representation of molecular docking of Omaveloxolone with KEAP1. **(C)** Molecular Docking Energy Scale. **(D)** The representative images show the levels of KEAP1, TH and β-actin by Western blotting. **(E)** Quantitative analysis showed that the expression levels of KEAP1 and TH. All data are presented as mean ± SEM, n = 3. The PD + Omaveloxolone group showed elevated expression of KEAP1 and TH compared to the PD group. ##P < 0.01 compared with the control group. *P < 0.05 and **P < 0.01 compared with the MPTP group.

Omaveloxolone, an emerging drug, was first approved by the FDA as the first Friedreich ataxia (FRDA) treatment (Subramony and Lynch, 2024). *In vitro* experiments, Omaveloxolone, as an agonist of Nrf2, attenuates the destruction of Nrf2, thereby rescuing the FRDA cell model (Abeti et al., 2018). To further explore the possibility of an anti-PD mechanism for Omaveloxolone, we constructed a network of drug targets for this drug (Figure 8A). This drug target network demonstrates that Omaveloxolone connects to 1 PD genes and 6 PPI partners (e.g., KEAP1 and Nrf2). The major regulator of redox homeostasis is Nrf2, the expression of genes containing antioxidant response element (ARE) sequences in the promoter region of Nrf2, including genes (e.g., NQO1 and HO-1) associated with the synthesis or use of GSH (Suzuki and Yamamoto, 2017). Keap1 acts as a substrate recognition subunit for E3 ubiquitin ligase and specifically targets Nrf2. The Kelch structural domain of KEAP1 has multiple protein contact sites that mediate the binding of KEAP1 and Nrf2. So we explored whether Omaveloxolone binds to KEAP1 by computer-simulated molecular docking. In Figure 8B, We found that Omaveloxolone is capable of binding to KEAP1 by computer-simulation methods. The docking scores for the combination between them are −6.80 kcal/mol respectively (Figure 8C). We similarly used Western blot to detect the expression of KEAP1 and TH (Figure 8D). We used three different concentrations (5 mg/kg, 10 mg/kg, 20 mg/kg) of Omaveloxolone to treat the PD mouse model. Our results show that Omaveloxolone treatment increases the expression of KEAP1 and prevented the downregulation of TH (Figure 8E) (*P* < 0.05). Among them, medium and high drug concentrations significantly increased the expression of KEAP1 in PD mice, and medium drug concentrations significantly prevented the downregulation of TH in PD mice (*P* < 0.01). Taking our results together, 10 mg/kg Omaveloxolone was the optimal concentration to exert anti-PD effects in the PD mouse model.

### 3.9 Omaveloxolone improves dopaminergic neuronal survival and reduces neurofunctional deficits in PD mice, with observed activation of the Keap1-Nrf2/ARE signaling pathway

**FIGURE 9:**
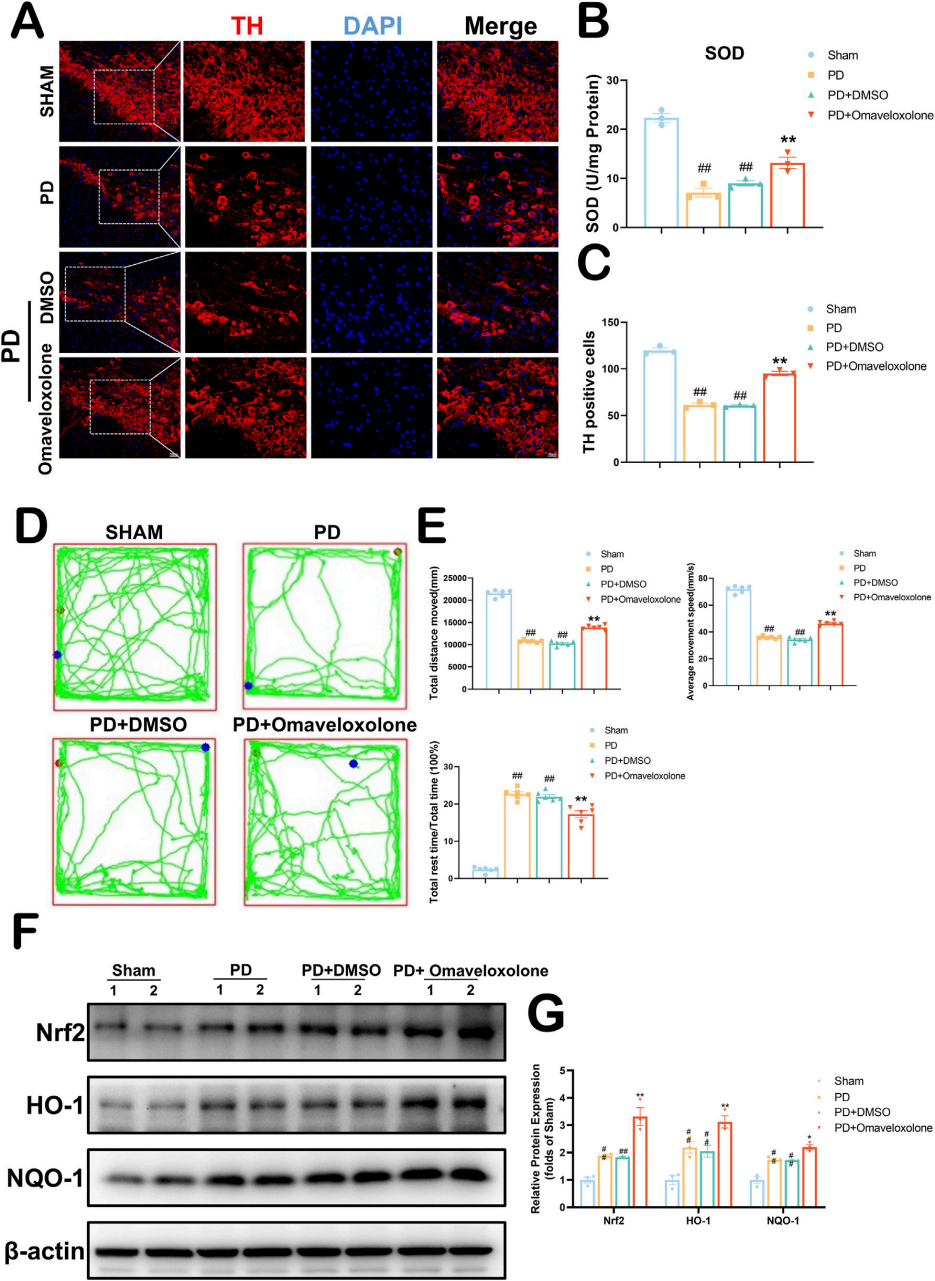
Omaveloxolone increases dopaminergic neuron expression to ameliorate neurologic deficits in PD via the Keap1-Nrf2/ARE signaling pathway. **(A)** Survival of dopaminergic neurons detected by TH fluorescence staining of the substantia nigra area. **(B)** Expression of SOD in the substantia nigra area by ELISA. All data are presented as mean ± SEM, n = 3. Omaveloxolone significantly elevated the expression of SOD in mice in the PD group. **(C)** Omaveloxolone significantly increases the survival of dopaminergic neurons in the substantia nigra area of PD mice. All data are presented as mean ± SEM, n = 3. **(D)** Neurological behavior of mice detected by Open field test. **(E)** The PD + Omaveloxolone group was significantly improved compared to the PD group in terms of total distance moved and average movement speed, and significantly less in terms of the proportion of rest time. All data are presented as mean ± SEM, n = 6. **(F)** The representative images show the levels of Nrf2, HO-1, NQO1 and β-actin by Western blotting. **(G)** Quantitative analysis showed that the expression levels of Nrf2, HO-1 and NQO1. All data are presented as mean ± SEM, n = 3. ##*P* < 0.01 compared with the control group. **P < 0.01 compared with the MPTP group.

*In vivo*, we used 10 mg/kg of Omaveloxolone to treat PD mice. In Figure 9A, We used immunofluorescence to detect TH expression in the mouse substantia nigra area. Our results show that the PD + Omaveloxolone group had significantly higher TH expression in the substantia nigra area than the PD group (Figure 9C, 91.51–101.83 vs. 52.60–70.06, *P* = 0.0003), representing a significant reduction in the loss of dopaminergic neurons. In Figure 9B, We examined the expression of Superoxide Dismutase (SOD) in the mouse substantia nigra area by ELISA kit. SOD is an important antioxidant enzyme in the first-line antioxidant defense mechanism of our body, in which SOD-1 belongs to the ARE gene of Nrf2 (Jomova et al., 2024). The PD + Omaveloxolone group significantly increased SOD expression levels in Parkinson’s disease mice relative to the PD group (Figure 9B, 10.76-16.92 vs3.29–10.95, *P* = 0.0019). We detected neurological deficits in mice by the open-field test (Figure 9D). In Figure 9E, There were no differences between the PD and PD + DMSO groups in terms of total distance moved, average movement speed, and proportion of rest time (*P* > 0.05). The PD + Omaveloxolone group was significantly improved compared to the PD group in terms of total distance moved and average movement speed, and significantly less in terms of the proportion of rest time (*P* < 0.01). To explore the mechanism of Omaveloxolone against PD, we examined the protein expression of Nrf2 and its regulated downstream genes (HO-1 and NQO1) (Figure 9F). We found that after Omaveloxolone treatment, the protein expression levels of Nrf2, HO-1 and NQO1 were significantly higher compared to the PD + DMSO group (Figure 9G, P < 0.01). Overall, Our evidence suggests that Omaveloxolone may enhance the survival of dopaminergic neurons in the substantia nigra of PD mice and reduce neurological deficits via the Keap1-Nrf2/ARE signaling pathway.

## 4 Discussion

Parkinson’s disease, the second most common neurodegenerative disease worldwide, continues to grow in prevalence (Ben-Shlomo et al., 2024). There is a notable deficiency in medications capable of fundamentally addressing the needs of patients with Parkinson’s disease. Currently, various dopamine replacement therapies are frequently employed; however, they fail to offer sustained symptom control. Consequently, the pursuit of novel therapeutic interventions remains imperative. In this study, we employed computational drug repurposing methodologies to identify potential therapeutic candidates for Parkinson’s disease, specifically highlighting Omaveloxolone and Cyproheptadine. Our approach utilized drug-target network analysis and predictive modeling, resulting in the identification of 176 drug candidates. Following an extensive review of existing literature and subsequent *in vitro* validation, Omaveloxolone and Cyproheptadine emerged as promising therapeutic candidates, demonstrating significant neuroprotective effects in PD models.

**FIGURE 10:**
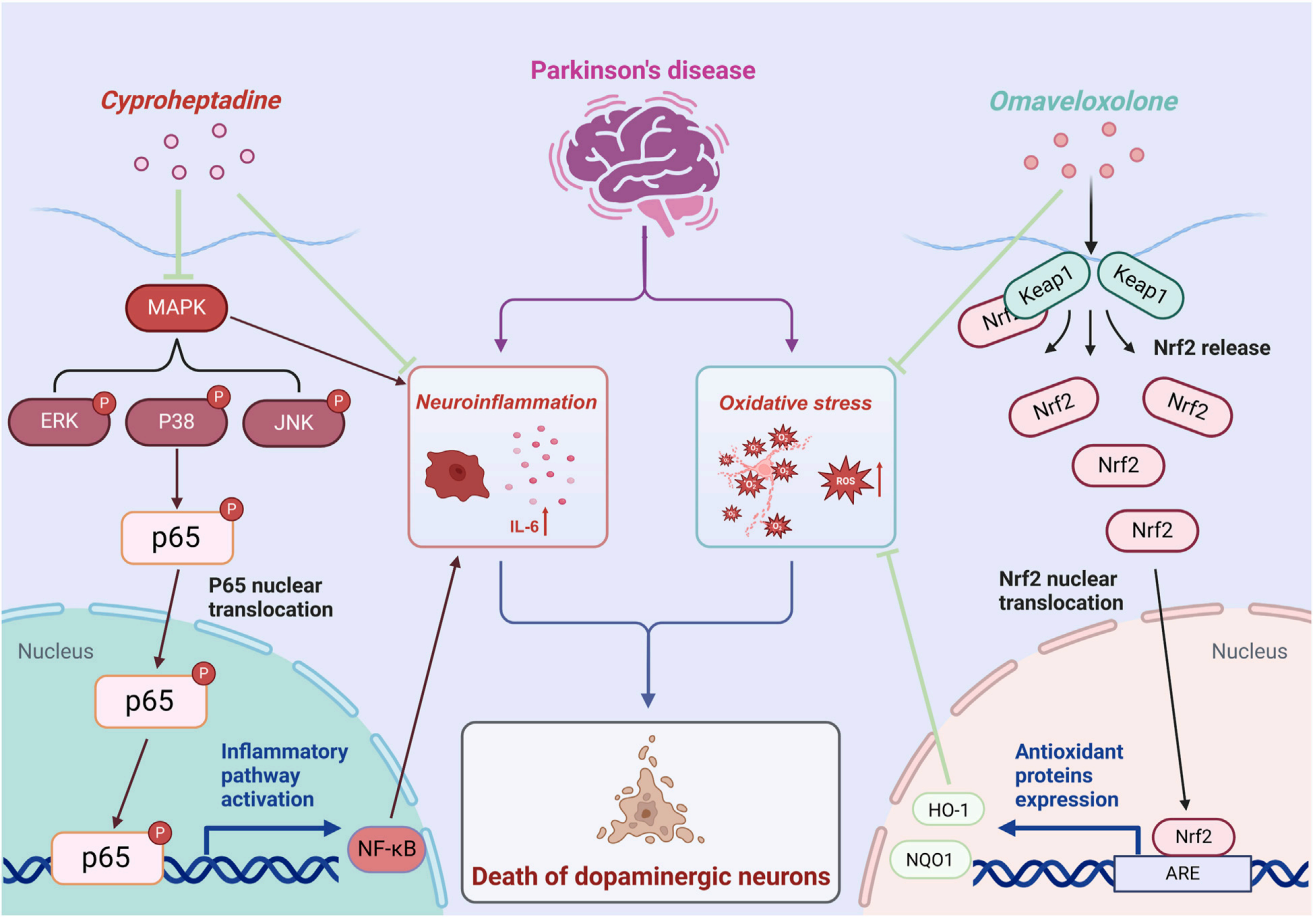
Mechanism of the anti-PD effects of Omaveloxolone and Cyproheptadine.

The prospective therapeutic benefits of Omaveloxolone and Cyproheptadine in Parkinson’s Disease are facilitated through their modulation of specific signaling pathways integral to the disease’s pathogenesis. Omaveloxolone, originally identified as a therapeutic agent for Friedreich’s ataxia (Subramony and Lynch, 2024), has shown promise as a neuroprotective compound in the context of PD. Our research suggests that Omaveloxolone activates the Keap1-Nrf2/ARE pathway, which is a pivotal regulator of the cellular antioxidant response (Ding et al., 2024). Our results suggest that Omaveloxolone’s neuroprotective effects may involve activating the Keap1-Nrf2/ARE pathway, promoting Nrf2 nuclear translocation, thereby activating ARE-driven genes and enhancing cellular defense against oxidative stress. Mitochondrial dysfunction and abnormal α-syn aggregation are central to PD pathology. As the key regulator of redox homeostasis, Nrf2 activation can effectively neutralize ROS caused by these issues in PD, thus slowing neuronal degeneration (Li et al., 2024; Vasquez et al., 2024). This is demonstrated by the elevated expression levels of Nrf2, HO-1, and NQO1 observed in our *in vivo* PD model treated with Omaveloxolone, indicating its protective role against neurodegeneration induced by oxidative stress. Additionally, our study explores the repurposing of cyproheptadine, a first-generation antihistamine (Badr and Naguy, 2022), for its potential therapeutic application in PD treatment. Our findings indicate that Cyproheptadine effectively inhibits the expression of IL-6, a pro-inflammatory cytokine, and prevents the downregulation of TH, a critical enzyme involved in dopamine biosynthesis. Microglia play a pivotal role in the pathogenesis of PD. Activated microglia release inflammatory mediators such as IL-6, which critically contribute to PD progression. Activation of the MAPK/NFκB pathway promotes microglia polarization to a pro-inflammatory phenotype, leading to more inflammatory mediator release and forming an inflammatory cascade (Qin et al., 2024). In our study, Cyproheptadine significantly inhibited MAPK/NFκB activation. It likely blocks this signaling amplification, reduces pro-inflammatory microglia polarization, and thus alleviates the chronic damage of neuroinflammation on dopaminergic neurons. The observed suppression of IL-6 and maintenance of TH expression by Cyproheptadine underscore its potential therapeutic role in alleviating neuroinflammation and preserving dopaminergic neurons in PD. Figure 10 presents a schematic diagram illustrating the intricate signal network modulated by Omaveloxolone and Cyproheptadine, thereby offering an in-depth perspective on the interaction of these drugs with cellular mechanisms and their potential to ameliorate the pathophysiology of Parkinson’s Disease.

The domain of computational drug repurposing has gained prominence as a transformative strategy in drug discovery, especially concerning intricate neurodegenerative disorders such as PD (Abdelrady et al., 2024). This study employs this innovative approach to identify Omaveloxolone and Cyproheptadine as potential therapeutic candidates for PD, thereby providing novel insights into treatment strategies. Conventional pharmacological interventions for PD predominantly concentrate on symptom management, including dopamine replacement therapy and deep brain stimulation (Hill et al., 2023). Although these methods offer temporary relief, they fail to target the fundamental neurodegenerative mechanisms, and their effectiveness is frequently constrained by adverse effects and the necessity for ongoing treatment modifications. Conversely, our computational approach has identified drugs with potential disease-modifying properties that may decelerate disease progression. Notably, these drugs have already received approval for other indications, indicating a potentially expedited transition to clinical application due to their established safety profiles. Unlike *de novo* drug development, which often faces high failure rates and long timelines (Paul et al., 2010), Omaveloxolone and Cyproheptadine show promise for Parkinson’s disease treatment. Omaveloxolone may offer neuroprotection through Nrf2 activation and enhanced antioxidant response, while Cyproheptadine could mitigate neuroinflammation, a crucial element in Parkinson’s disease. The *in vitro* and *in vivo* validation of these drugs in our study substantiates their potential progression to clinical trials, thereby presenting a novel avenue for therapeutic intervention in Parkinson’s Disease.

Nevertheless, it is important to acknowledge certain limitations inherent in our study. Firstly, while our compilation of databases pertaining to Parkinson’s disease and associated medications was derived from several prominent and authoritative sources, it remains incomplete. Secondly, our drug prediction database may be constrained and lacks comprehensive authoritative evaluation. Furthermore, during the final screening of nine drugs, Ephedrine was unavailable, precluding the possibility of evaluating its medicinal properties. We selected Cyproheptadine and Omaveloxolone as representative candidates for evaluating anti-Parkinson’s disease pharmacologic action. Due to constraints related to cost and time, our study focused exclusively on these two drugs. It is important to note that this selection does not imply that other drugs lack potential value for testing. Notwithstanding these limitations, the study’s merits are found in its novel use of computational methodologies and the recognition of potential therapeutic candidates, necessitating additional preclinical and clinical assessment. Although our data support the association between Omaveloxolone and activation of the Keap1-Nrf2/ARE pathway, the necessity of this pathway for its neuroprotective effects hasn't been fully validated. Future studies using Nrf2-specific inhibitors will help strengthen the causal inference. Our study used the MPTP-induced PD model, which mimics acute dopaminergic neuron loss and neuroinflammation but lacks α-syn aggregation seen in human PD. Though reproducible and rapid in inducing motor deficits, its inability to reproduce α-syn pathology limits direct translation to human PD. Future studies using α-syn pre-formed fibril (PFF) models or transgenic α-syn overexpression models (e.g., A53T-SNCA mice) could verify the neuroprotective effects of Omaveloxolone and Cyproheptadine in an α-syn context. Combining MPTP with α-syn-based models may offer a more comprehensive platform for evaluating therapeutics targeting both neurodegeneration and protein aggregation. While our results show consistent statistical significance across multiple endpoints (P < 0.05 or lower), we acknowledge that future studies with larger samples could further validate these findings and enhance their translational relevance, especially considering the inherent variability in behavioral and molecular outcomes. Despite these limitations, it is noteworthy that this study is the first to integrate Parkinson’s disease targets with a database of FDA-approved drugs for the purpose of drug repurposing and screening. The reliability of network-based computational repurposing was substantiated through both *in vivo* and *in vitro* experiments. This methodology facilitates the identification of additional potential therapeutic agents for Parkinson’s disease.

In conclusion, the efficacy of our computational drug repurposing methodology in identifying these candidates underscores the strategy’s effectiveness in the realm of drug discovery. By utilizing existing pharmaceuticals with established safety profiles, this approach facilitates the accelerated development of novel therapies for Parkinson’s Disease, thereby diminishing the time and financial resources typically required in conventional drug development processes. Furthermore, this strategy not only offers a more direct pathway to clinical application but also broadens the scope for investigating the diverse therapeutic potential inherent in existing drugs.

## Data Availability

The datasets presented in this study can be found in online repositories. The names of the repository/repositories and accession number(s) can be found in the article/Supplementary Material.
